# It is time to focus on an underestimated epidemic

**DOI:** 10.1590/S1980-57642014DN84000001

**Published:** 2014

**Authors:** Gladys E Maestre, Ricardo Nitrini

Contemporary dementia research has spurred remarkable interest and endogenous efforts by clinical and research teams in Latin America and the Caribbean (LAC). The combination of unprecedented high burden of dementia, low resource settings, and wide acceptance of age-related cognitive changes has put thousands of individuals and families in a vulnerable situation that requires immediate attention. This issue of **Dementia *&* Neuropsychologia** is dedicated to cognitive impairment and dementia in LAC, and presents a timely series of fourteen reviews and original articles from Argentina, Brazil, Chile, Colombia, Costa Rica, Cuba, Dominican Republic, Ecuador, Peru, Trinidad and Tobago, and Venezuela. Many of the topics have been extensively examined by clinicians and scientists all over the world, but the focus on LAC is just emerging.

Many of the articles in this issue take an interdisciplinary approach, and several are the product of international collaborations that have facilitated epidemiological studies. The review paper by **Toledo et al**. plots the recent growth of dementia research in Brazil, evidence that LAC is making significant contributions to our understanding of dementia and related topics, due to recent investments in graduate and postgraduate education. National expertise in dementia research, and national programs that provide care and support to patients and caregivers, are clearly stronger than ever, and more international collaborations will undoubtedly accelerate the generation of knowledge. However, even greater effort is needed to apply that growing knowledge to alter the course of the not-so-hidden dementia epidemic in LAC.

Although cardiovascular disease is the leading cause of death worldwide, infectious diseases are still dominant in LAC and other developing regions of the world. Traumatic brain injury, resulting from violence, traffic accidents, etc., is also common in LAC, resulting in particular vulnerability to age-related dementia. Papers in this issue from Argentina (**Bartoloni et al. and Arizaga et al.**), Cuba (**Llibre-Rodriguez**), Chile (**Fuentes and Albala**), Brazil (**Dozzi-Brucki and Nitrini**), and Colombia (**Amaya et al**.) show that prevalence of dementia is as high in LAC as in developed countries. Studies of risk factors suggest that low cognitive reserves, related to low educational and socioeconomic levels, are a major risk factor in the region.

Standardized diagnostic tools for assessing cognitive and daily performance, which take into account cultural differences and low education levels, have been developed for use in LAC. However, biochemical and radiological markers need to be identified to facilitate early and accurate diagnosis of cognitive impairment. In this issue, **Baboolal et al**. discuss a serum screening biomarker for dementia that could fundamentally alter our approach to healthcare maintenance of the elderly. **Del Brutto et al.** describe development of a screening test that reflects global cortical atrophy. **Cesar et al.** evaluate a simple test for depression, a common co-morbidity in cognitive impairment, which can be used in epidemiologic studies.

Despite the ever-increasing wealth of upper income groups in many LAC countries, the majority of people are poor. Dementia has many costs, not least of which is the suffering of family members and informal caregivers. **Custodio et al**. in Peru, and **Medrano et al.** in the Dominican Republic, provide objective measurements of the burden of dementia among non-professional caregivers. Scientists, clinicians and advocacy groups are working to develop the human resources and infrastructure in LAC to relieve this burden, as reported by **Miranda-Valverde et al**. in Costa Rica, and **Gonzalez et al.** in Venezuela. However, a report from Chile by Fuentes and Albala indicates that, despite increased awareness by the general public and the current efforts to demonstrate the need for action, there has been insufficient response from the state to the diagnostic and therapeutic requirements of dementia patients and caregivers.

It is hoped that this issue of **Dementia *&* Neuropsychologia** will provide readers with an overview of the current developments in dementia research and care in LAC, although it is not possible to cover every aspect of the field, or every research group in the region. It is also hoped that this dedicated issue will inspire new generations to engage in research and healthcare for cognitive impairment and dementia in LAC.

**Gladys E. Maestre** **Ricardo
Nitrini**

